# The correlation between cognitive performance and retinal nerve fibre layer thickness is largely explained by genetic factors

**DOI:** 10.1038/srep34116

**Published:** 2016-09-28

**Authors:** Eneh Jones-Odeh, Ekaterina Yonova-Doing, Edward Bloch, Katie M. Williams, Claire J. Steves, Christopher J. Hammond

**Affiliations:** 1 Department of Twin Research and Genetic Epidemiology, King’s College London, London, United Kingdom; 2 Ophthalmology, King’s College London, London, United Kingdom

## Abstract

Retinal nerve fibre layer (RNFL) thickness has been associated with cognitive function but it is unclear whether RNFL thinning is secondary to cortical loss, or if the same disease process affects both. We explored whether there is phenotypic sharing between RNFL thickness and cognitive traits, and whether such sharing is due to genetic factors. Detailed eye and cognitive examination were performed on 1602 twins (mean age: 56.4 years; range: 18–89) from the TwinsUK cohort. Associations between RNFL thickness and ophthalmic, cognitive and other predictors were assessed using linear regression or analysis of variance models. Heritability analyses were performed using uni- and bivariate Cholesky decomposition models. RNFL was thinner with increase in myopia and with decrease in disc area (p < 0.001). A thicker RNFL was associated with better performance on mini mental state examination (MMSE, F(5,883) = 5.8, p < 0.001), and with faster reaction time (RT, β = −0.01; p = 0.01); independent of the effects of age, refractive error and disc area (p < 0.05). RNFL thickness was highly heritable (82%) but there was low phenotypic sharing between RNFL thickness and MMSE (5%, 95% CI: 0–10%) or RT (7%, 95% CI: 1–12%). This sharing, however, was mostly due to additive genetic effects (67% and 92% of the shared variance respectively).

Cognitive function has been defined as the ability to appropriately identify and manage complex external or internal stimuli, and encompasses many mental abilities and processes, including decision making, memory, attention, and problem solving^1^. The retinal nerve fibre layer (RNFL) has been shown to be thinner in people with memory loss and cognitive decline in certain neurodegenerative conditions such as Alzheimer’s disease (AD) and multiple sclerosis (MS)^2^. The retina forms as a functional extension of the central nervous system (CNS), developing as a result of diencephalic evagination of pluripotent cells during embryogenesis^3^ and shares similar morphological properties with other neurons in the CNS^4^. These properties allow the opportunities for studying the retina as a model of the CNS, and provide window for accessible and direct observation of the CNS using ocular imaging techniques such as optical coherence tomography (OCT).

OCT is now the most commonly used optical imaging technique for structural assessment of the retina, allowing for accessible, inexpensive and non-invasive *in vivo* quantitative measurement of the optic nerve head (ONH) and RNFL. With similar principles to that of ultrasound, OCT uses low-coherence interferometry to measure optical backscattering of light from the retina and a reference mirror and calculates RNFL thickness (RNFLT) from generated two-dimensional data sets.

At a population level, a thinner RNFLT has been associated with lower cognitive performance^5^,^6^,^7^ in cross-sectional analysis, although the Erasmus Rucphen Family (ERF) Study only found this association in younger individuals^6^ and the association was reversed in the older Lothian Birth Cohort Study when corrected for IQ at age 11^5^. It is also well-described in populations with mild cognitive impairment^2^. However it is unclear whether RNFL thinning is secondary to cortical loss (retrograde damage), or if the same disease process affects both brain and retina.

A twin and a family study have demonstrated heritability of the RNFLT^8^,^9^, but to date, to our knowledge, no studies have explored whether this phenotypic sharing is due to shared genetic or environmental factors.

The aim of this large twin study was to examine whether the RNFLT measured by OCT was associated with cognitive function and whether this association was driven by shared genetic factors. A further objective of this study was to explore associations between RNFLT and age, sex, spherical equivalent (SphE), and other factors known to influence glaucoma such as intraocular pressure (IOP) and optic disc size.

## Results

### Association analysis

**Figure 1:**
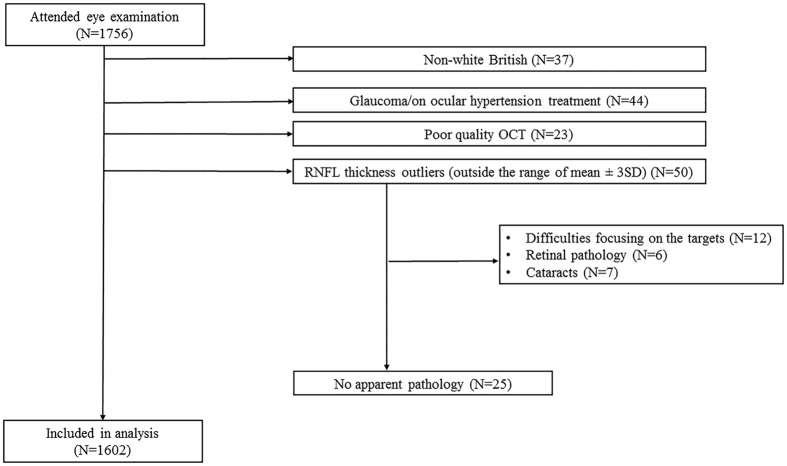
Consort diagram of the study. Legend: This figure presents a consort diagram of the study showing reasons for exclusion from the study.

We initially explored associations between RNFL thickness and various ophthalmological and cognitive measures (Table 1). 1602 twins of 908 families (mean age 56.4 years; range 18–89) from the TwinsUK cohort were included (Fig. 1). There was no difference between monozygotic (MZ) and dizygotic (DZ) twins except for the following variables (p < 0.05): age, reaction time (RT), MMSE score and recall (RC) (Table 1). The MZ twins were on average slightly younger than DZ twins (55 versus 59 years of age) and had better reaction times, MMSE score and recall. When adjusted for age, however, the three cognitive parameters did not differ between MZ and DZ twins. The inferior quadrant had the highest RNFL thickness followed by superior, nasal and temporal quadrants. The differences in thickness between quadrants were statistically significant (p < 1 × 10^−4^). All of the RNFL parameters were approximately normally distributed.

Table 2 summarises the univariable (A) and multivariable (B) results from association analysis for the quantitative predictors. Increase in age was associated with decrease in RNFL thickness for the average of the four quadrants and in each quadrant individually (p < 0.001). Education was nominally associated with average RNFL thickness and with the inferior and temporal quadrants in the univariable analysis only (p < 0.05). Increase in SphE (more hyperopic refraction) and in disc area (larger optic discs) were strongly associated (p < 0.001) with increase in RNFL thickness for the average of the four quadrants and in each quadrant, except for SpheE in the temporal quadrant (p = 0.41). Disc area remained strongly associated (p < 0.001) in the multivariable analysis with the average of the four quadrants and in all the quadrants separately, while age and SphE were associated with RNFLT for all but the temporal quadrant (Table 2). There was no association between sex, index of multiple deprivation (IMD) or IOP corrected for central corneal thickness (CCT) and RNFLT (Table 2).

None of the cognitive parameters were associated with SphE, education or IMD, independently of age (p < 0.05). A faster reaction time was associated with a thinner RNFL thickness (p < 0.001) and this stayed significant in the multivariable model for average RNFL (p = 0.01), and for the inferior and temporal quadrants (p = 0.01 and p = 0.04 respectively). As cataract surgery has been reported to improve reaction times^10^, we compared the reaction time in the 63 pseudophakic participants to that of 126 age-matched individuals without cataract surgery (case:control ratio of 1:2). There was no difference between the two groups (mean of 630 ms and 632 ms respectively, p = 0.8). Cataract surgery status was associated with RNFL thickness in univariable analysis (β = −3.29, p = 0.02), but not in the multivariable analysis (likely to due to the association between cataract surgery and age).

There were significant differences in RNFL thickness between MMSE groups (p_trend_ < 0.003). The one-way ANOVA results for MMSE were as follows: average RNFL – F(5, 883) = 5.8, p < 0.001; Superior quadrant – F(5, 883) = 4.0, p = 0.001; Inferior quadrant – F(5, 883) = 5.9, p < 0.001; Temporal quadrant – F(5, 883) = 2.5, p = 0.02; Nasal quadrant – F(5,883) = 2.8, p = 0.02. These differences were still significant after taking age, SphE and disc area into account, as determined by MANOVA for all but the temporal quadrant: average RNFL – F(5,615) = 4.2, p = 0.001; Superior quadrant – F(5,615) = 3.1, p = 0.01; Inferior quadrant – F(5,615) = 3.8, p = 0.002; Nasal quadrant – F(5,474) = 2.6, p = 0.02. Recall was associated with RNFL thickness only in the inferior quad-rant –F(5,886) = 5.2, p = 0.01, however this effect was not independent from the effects of age, SphE and disc area F(2,618) = 2.9, p = 0.06. No statistically significant association was detected between RNFL thickness and verbal fluency (VF) (data not shown).

### Heritability analysis

The results of the univariate heritability models are reported in Table 3. In all cases the best fitting model was the AE (addictive genetic effects/unique environment) model. As can be seen from the table, RNFL thickness is highly heritable with additive genetic effects accounting for 65% (Nasal quadrant) to 83% (the average of the four quadrants) of variance. As pointed above, due to the fact that the MZ twins were younger than the DZ twins, there were differences between the two groups in terms of MMSE, RC and RT. For this reason we adjusted those predictors for the effect of age prior to the heritability analysis. The measurements of cognitive function were moderately heritable (MMSE = 32%, RT = 44% and VF = 48%), with the exception of recall which showed low heritability (19%).

**Figure 2:**
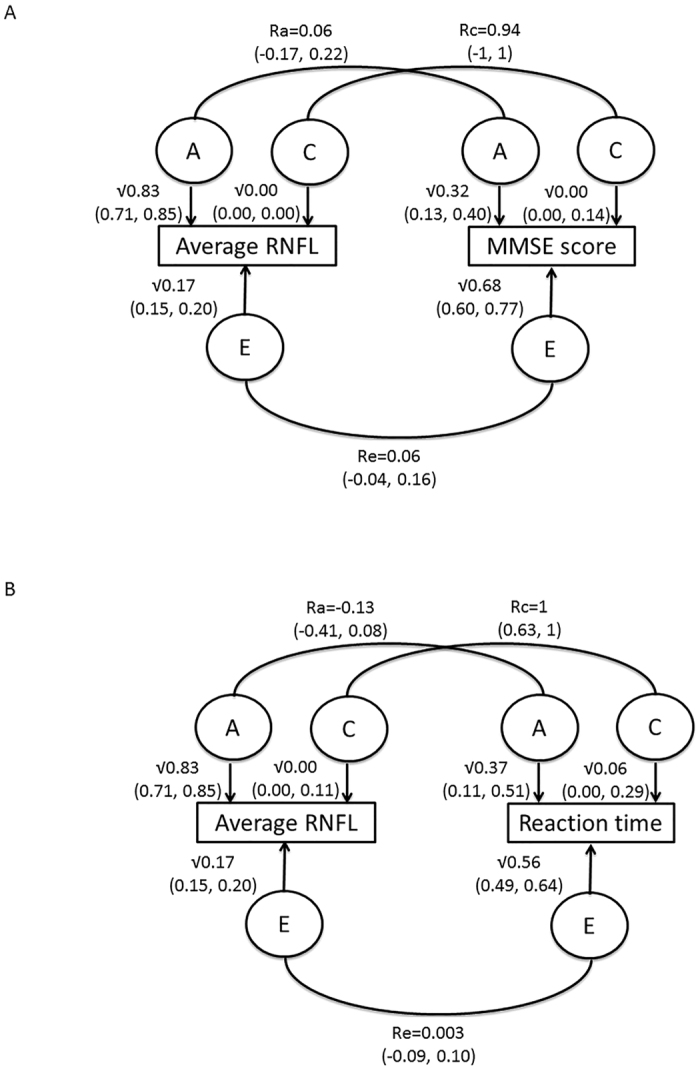
Bivariate heritability model showing the amount of shared genetic variation for average RNFL thickness and MMSE (**A**) and Reaction time (**B**). Legend: This figure presents the bivariate heritability models for RNFL thickness and the associated cognitive parameters. All traits were adjusted for age prior to the analysis. RNFL – retinal nerve fibre layer; MMSE – mini-mental state examination; A – additive genetic factors; C – common/shared environmental factors; E – unique environmental latent factors; Ra – genetic correlation; Rc – common environment correlation; Re – unique environment correlation.

When taking into account the effect of age, RNFL thickness shared 5% (95% CI: 0−10%) of phenotypic variance with MMSE and 7% with RT (95% CI: 1–12%) (Fig. 2). Additive genetic effects accounted for 67% (95% CI: 61–81%) and 87% (95% CI: 82–99%) of the shared variance for MMSE and RT respectively and rest was explained by environmental factors. Adjusting for SphE and disc area did not substantially change the results: phenotypic sharing between MMSE and RNFL was 8% (95% CI: 2–15%) and of those 93% (72–96%) was due to additive genetic factors; phenotypic sharing between RT and RNFL was 11% (95% CI: 4–7%) and of those 82% (75–94%) was due to additive genetic factors.

## Discussion

This study, based on an unselected twin population, has confirmed an association between thinner RNFL and lower cognitive scores, and suggests this phenotypic sharing is largely due to shared genetic factors. This raises the possibility that the processes involved in cognitive decline and ultimately dementia are similar to those resulting in loss of RNFL, and the latter could therefore be a biomarker of dementia. However, the degree of phenotypic sharing (8% for MMSE score and 11% for reaction time, when adjusted for age, refractive error and optic disc size) is relatively small, and given other factors such as glaucoma influence RNFL thickness, it remains to be seen if this will have enough specificity and sensitivity to act as a biomarker of disease progression in dementia.

Our findings are similar to another UK-based aging cohort from the Norfolk EPIC study by Khawaja *et al*.^7^, which showed significant, albeit weaker, associations with MMSE and also the Hopkins Verbal Learning Test (testing recognition, learning and memory) and the National Adult Reading Test (NART, testing premorbid intelligence). Similarly, the Dutch ERF study^6^ showed better cognitive function associated with thicker RNFL across various measures, with 2.8% of cognitive scores explained by RNFLT, although somewhat surprisingly the strongest effects seemed to be in the younger age groups (below 40 years). Some of these differences may be due to measurement techniques, and these studies used HRT (Heidelberg Retina Tomograph) and scanning laser polarimetry (the GDx VCC) rather than the current gold standard used in this study, of OCT. The Lothian study^5^ using OCT in only 96 subjects aged 73 years, found that while cognitive decline seemed to be associated with thinner RNFL, when corrected for IQ at age 11, those with thicker RNFL had worse cognition, again somewhat counterintuitively. We do not have historic IQ data on the TwinsUK cohort, but did not observe any relationship between the educational and socio-economic status and either RNFL or the cognitive parameters in this study. The cognitive measures used in each study differed, which may also explain some of the differences; we selected MMSE, RT and VF because these tests are commonly used in clinical settings^11^,^12^,^13^,^14^.

Our results showed that the average RNFL was significantly thinner with increasing age, lower spherical equivalent (i.e. myopia) and with smaller optic disc size. Similar findings have been confirmed in other studies^15^,^16^ and an inverse relationship between RNFLT and SphE identified in a small twin study of 50 pairs^8^. We found the phenotypic sharing between RNFL and cognition was greater when these factors were accounted for, suggesting future studies examining RNFLT variation should measure refractive error or axial length, which is was measured in the Khawaja *et al*. study^7^ but is not commonly done in non-ophthalmic studies, in addition to adjusting for age and disc size (which is measured by OCT).

Our community-based study confirms the relationship between RNFL and cognitive decline in clinic-based studies which found a thinner RNFL in patients even with mild cognitive impairment^17^,^18^,^19^,^20^. In particular, the inferior quadrant has been proposed to be more susceptible to neuronal loss^2^,^14^,^21^,^22^. Certainly in our data, and others^5^, the inferior quadrant showed the strongest relationships with cognitive tests, although as the quadrant with the thickest RNFL it is unsurprising that the effect size is largest in ANOVA analyses, but despite this, the inferior (along with the temporal) quadrant was the only quadrant which remained significantly associated with reaction time when age, spherical equivalent and disc size were included in the multivariable model. Histological studies have shown significant correlation between retinal layers determined by OCT and retinal histology^23^ demonstrated reduced RNFL thickness in patients with AD when compared with normal controls^24^, however individual quadrants of the RNFL were not examined separately. It is unclear why the inferior RNFL might be preferentially affected but it may be due to the fact that nerve fibers with larger diameter atrophy more rapidly and those fibers are more abundant in inferior and superior quadrants^25^.

RNFL thickness was shown in this large twin study of 730 pairs to be highly heritable, while MMSE score and RT were moderately heritable with additive genetic effects accounting for 82%, 32% and 44% of variance respectively. These results are in line with previous twin studies^8^,^26^, and as expected, higher than found using other family-based study designs^9^. Many quantitative traits that can be measured accurately are highly heritable, such as refractive error and height, and both these traits appear to be influenced by hundreds of common genetic variants^27^,^28^,^29^. Genome-wide association studies (GWAS) of RNFL are in progress, and we await with interest whether genes or pathways identified in these analyses are common to dementia GWAS.

This study is not without its limitations. First, as the TwinsUK cohort consists mainly of white British middle-aged females (mean 56.2 years, SD 15.4) we were not able to look at gender or ethnicity differences. However, our baseline measurements of average RNFL thickness (96.2 ± 9.1 μm) are similar to those obtained in healthy controls of other studies^11^,^12^,^30^ that have investigated the cognitive associations of RNFL thickness using OCT (91.5 ± 7.1 μm), (98.6 ± 1.67 μm), (94.3 ± 11.3 μm) respectively, and quadrant thickness distribution (ISNT rule: inferior RNFL thickest, followed by superior, nasal and temporal) comparable to those obtained in similar studies^5^,^14^,^31^. We found no differences between genders for RNFL (and with 183 males the sample size in not insignificant). We therefore believe that these data are generalisable to the wider European population. Second, we do not have longitudinal cognitive or RNFL data on these subjects, so causality cannot be applied to any of the cross-sectional analyses, and these results may still be due to residual confounding by age, or refractive error, not fully corrected in linear models, or other confounders. Third, due to the low phenotypic sharing we observed, the bivariate heritability results were of borderline statistical significance, and larger studies might be needed for further analyses, including examining the amount of variance explained by known genetic variants from dementia genome-wide association studies.

In conclusion, we have shown RNFL thickness to be associated with MMSE and reaction time cognitive performance in a healthy adult population, and the phenotypic sharing was largely due to shared genetic factors. However, given the relatively low phenotypic sharing, RNFL thickness is probably a poor predictor of cognitive decline in an unselected population, although it remains to be determined in longitudinal studies, combining several and different cognitive tests, how predictive RNFL thickness might be. Further genetic studies may provide insight into shared pathophysiology between loss of RNFL and cognitive impairment.

## Methods

### Subjects

The TwinsUK cohort is a volunteer twin registry of 11,000 British adults, recruited from the general population through national media campaigns in the United Kingdom, and representative to the broader population in terms of disease-related and lifestyle characteristics^32^. All subjects were unaware of the hypotheses in this study at the time of enrolment. The studies in the TwinsUK cohort were performed with the approval of the Guys and ST Thomas’ Ethics Committee, and participants signed a written informed consent in accordance with the Declaration of Helsinki. All methods were performed in accordance with the relevant guidelines and regulations.

Between August 2014 and March 2016, 1756 twins from 974 families underwent detailed eye examination which included RNFL thickness measurements using spectral domain optical coherence tomography (Optovue iVue system, Fermont, California), non-cycloplegic autorefraction, non-contact IOP and central corneal thickness (CCT) measurements (Visionix120, The Luneau Technology group) of both eyes. Of the 1756 individuals, 154 were excluded, leaving 1602 twins of 908 families in the final analysis (Fig. 1). The reasons for exclusion were as follows: 37 individuals were of non-white British ethnicity; 44 individuals had self-reported diagnoses of glaucoma or were on treatment for ocular hypertension; 23 had poor quality OCT scans; and 50 individuals were outliers (fell outside the range of mean ± 3 SD) in terms of RNFL thickness (either average RNFL or in one of the 4 quadrants). For 25 out of the 50 outliers there was no obvious explanation why their measurements were so different to the mean and the scans for those individuals were deemed of good quality by an ophthalmologist. Of the remaining outliers, 12 individuals were described as having difficulty with target fixation, 6 presented with retinal pathology and 7 reported having cataracts in the eye/eyes with the aberrant RNFL measurement. From the 1602 twins included in the study, 63 (3.9%) reported having had cataract surgery previously.

In addition, data on age, sex, education (primary, secondary and higher) and socio-economic status (Index of Multiple Deprivation - IMD) were also collected.

### Cognitive assessment

As part of the visit, the twins also underwent cognitive assessment including the standardised mini-mental state examination (MMSE), verbal fluency (VF), and reaction time (RT). Recall (RC), a subtest included in the MMSE was extrapolated as an individual test and results obtained from this subtest were analysed separately. Cognitive data was available for 1593 individuals for the VF, MMSE and recall tests, and 1563 individuals for the RT test.

The MMSE is used widely in clinical settings as a standard screening tool for global measurement of cognitive function^33^. It contains 20 subtests with a maximum score of 30 and helps facilitate detection of cognitive impairment or change in mental status^34^. We decided to analyse RC scores separately as it has been shown to provide a good measure of episodic memory, and some other components of the full MMSE may not adequately discriminate healthy individuals in a population with mild cognitive impairment^35^.

The verbal fluency tasks assesses the speed and ease at which individuals can access words from memory^36^, and performance has been associated with both temporal and frontal lobe activity^37^,^38^. VF was tested by examiners instructing participants to name as many words beginning with a given letter of the alphabet, excluding names of people or places, and a further test required participants to name as many animals beginning with any letter. Participants were given 1 minute for each component of the VF test and were marked out of 7 for each task, where 0 = 2 answers or less and 7 = 17 answers or more. The scores from the two tasks were summed and individuals were categorised into two groups (normal performance, low performance) with cut-off score of 11.

RT was measured using the ‘Deary-Liewald Reaction Time Tester’ on a Windows 7 computer and was measured in milliseconds^39^. The test comprises of two components, the simple reaction time test (pressing a key anytime a target appears) and the choice reaction time test (pressing a key corresponding to 1 of 4 boxes where a target appears). Participants had 8 practice trials and 20 experimental trials for each component of the test. In the present study we analysed the mean of the two tests.

### Statistical Analysis

#### Association analysis

Comparisons of means and proportions for all variables between Monozygotic (MZ) and Dizygotic (DZ) twins or between RNFL quadrants were performed using two-sample two-tailed t-tests or z-tests, assuming equal variance. Associations between RNFL (either average or per quadrant) and age, sex, SphE, IOP and reaction time was assessed using univariable linear regression analyses, followed by multivariable linear regression model for factors showing significant (p < 0.05) association in the univariable model. Independent variables were identified using stepwise backwards procedure with threshold for removal set at 0.05. In all regression models family structure was taken into account.

Associations between RNFL thickness (either average or per quadrant) and MMSE, verbal fluency and recall were assessed using one-way ANOVA, followed by MANOVA that included age, SphE and disc area into the model. For these analyses only one twin per pair was selected at random. As there was strong correlation between the two eyes, we used the mean measurements of the two eyes for all eye phenotypes. All analyses were carried using STATA14 statistical package (www.stata.com).

#### Modelling of Heritability

Heritability analyses were performed on 730 twin pairs (438 monozygotic and 292 dizygotic) as data was missing on 178 co-twins. Zygosity was determined by a standardised questionnaire and confirmed using genome-wide single nucleotide polymorphism genotyping data or DNA short tandem repeat fingerprinting.

Univariate and bivariate Cholesky decomposition heritability models of RNFL thickness and the associated cognitive phenotypes were calculated using maximum likelihood structural equation twin modelling implemented in the OpenMx package (http://openmx.psyc.virginia.edu). The univariate method separates phenotypic variation in additive (A) genetic factors and common/shared (C) or unique environmental (E) latent factors, while bivariate model defines the amount of shared genetic variation that explains a given amount of shared phenotypic variance between two or more traits. In cases where the intraclass correlation coefficient for MZ twins was more than twice that of the DZ twins, ADE models were also computed (D stands for Dominant factors). The goodness of fit of the full and reduced ACE/ADE models were compared with the observed data and the best fitting model was selected. Two different bivariate models were explored. In Model 1 RNFL thickness was adjusted for age prior to the analysis, while in Model 2 RNFL was also adjusted for SphE and disc area in addition to age. In both models the cognitive traits were adjusted for age only.

## Additional Information

**How to cite this article**: Jones-Odeh, E. *et al*. The correlation between cognitive performance and retinal nerve fibre layer thickness is largely explained by genetic factors. *Sci. Rep.* **6**, 34116; doi: 10.1038/srep34116 (2016).

## Figures and Tables

**Table 1:** Characteristics of the study population.

	All subjects (N = 1602)		MZ twins (N = 947)		DZ twins (N = 655)	
	Mean (SD)	Range	Mean (SD)	Range	Mean (SD)	Range
Age(years)*	56.4 (15.2)	18.2–89.8	54.5 (16.0)	18.2–88.8	59.1 (13.4)	19.1–89.8
Average RNFL (μm)	96.8 (9.2)	66.6–123.2	97.1 (9.1)	70.1–123.2	96.3 (9.3)	66.6–120.3
Superior (μm)	118.2 (13.4)	74.7–158.9	118.3 (13.4)	74.4–155.5	118.1 (13.5)	74.7–158.9
Nasal (μm)	75.1 (10.8)	40.0–112.5	75.3 (10.9)	40.0–112.5	74.7 (10.7)	46.0–110.9
Inferior (μm)	121.4 (13.7)	73.3–167.5	122.0 (13.6)	76.4–167.5	120.5 (13.8)	73.3–159.7
Temporal (μm)	72.4 (8.7)	43.6–102.3	72.8 (8.9)	43.6–102.3	71.8 (8.4)	48.5–101.1
SphE (D)	−0.1 (0.1)	−11.3–9.3	−0.2 (2.4)	−11.3–7.5	0.1 (2.3)	−9.3–9.3
IOP (mmHg)	13.4 (2.7)	6.7–23.8	13.4 (2.7)	7.0–23.8	13.5 (2.7)	6.6–22.5
Corneal Thickness (μm)	538 (38.3)	221.2–752.3	537.7 (38.3)	221.3–752.3	538.4 (38.4)	343.0–745.6
Reaction time (ms)*	586.7 (87.9)	341.1–928.3	566.7 (101.7)	341.1–967.9	583.7 (95.5)	368.7–1049.5
	%		%		%	
Gender						
Female/Male	88.6/11.4		87.9/12.1		90.9/9.1	
Education*						
Primary	19.1		16.4		22.4	
Secondary	53.4		51.5		55.7	
Higher	27.5		32.0		21.9	
IMD						
Q1	6.9		7.4		6.2	
Q2	12.0		11.8		12.2	
Q3	18.6		19.1		18.1	
Q4	26.9		27.5		26.3	
Q5	35.7		34.3		37.3	
MMSE score*						
≤25	1.3		0.8		2.1	
26	2.1		2.3		1.9	
27	5.0		4.0		6.5	
28	12.5		11.7		13.5	
29	29.8		27.9		32.6	
30	49.2		53.2		43.3	
Recall*						
≤1	5.4		4.1		3.0	
2	21.9		20.3		10.0	
3	72.6		75.6		68.4	
Verbal fluency						
<11	16.4		16.6		16.0	
≥11	83.6		83.4		84.0	

Legend: This table shows summary data for the different variables studied for the whole sample population and by zygosity status. The ^*^denotes variables which were significantly different (p < 0.05) between monozygotic (MZ) and dizygotic (DZ) twins. RNFL –retinal nerve fibre layer; μm – micrometers; SphE – spherical equivalent; IOP – Intraocular pressure. ms – milliseconds, IMD – index of multiple deprivation from Q1 – bottom 20% most deprived to Q5 – top 20% most affluent.

**Table 2:** Univariable and Multivariable Linear Regression Analyses of RNFL thickness and ophthalmic, wcognitive and other predators.

	(A) Univariable														
	Average RNFL			Superior quadrant			Nasal quadrant			Inferior quadrant			Temporal quadrant		
	beta	se	p	beta	se	p	beta	se	p	beta	se	p	beta	se	p
Age	−0.15	0.02	<0.001	−0.21	0.03	<0.001	−0.13	0.02	<0.001	−0.19	0.03	<0.001	−0.07	0.02	<0.001
Sex	0.46	1.56	0.76	0.32	2.17	0.88	−0.85	1.89	0.65	1.61	2.48	0.52	0.77	1.36	0.57
Education	0.97	0.46	0.04	1.06	0.68	0.12	0.43	0.54	0.43	1.33	0.65	0.043	1.05	0.42	0.01
IMD	0.19	0.24	0.42	−0.05	0.35	0.90	0.25	0.28	0.37	0.24	0.34	0.47	0.32	0.22	0.14
SphE	1.14	0.13	<0.001	1.49	0.19	<0.001	1.43	0.15	<0.001	1.54	0.21	<0.001	0.12	0.15	0.41
IOP	−0.10	0.15	0.50	−0.24	0.22	0.13	0.10	0.17	0.61	−0.06	0.21	0.77	−0.22	0.13	0.09
IOPc	−0.06	0.11	0.59	−0.01	0.16	0.93	0.02	0.12	0.85	−0.15	0.16	0.33	−0.09	0.10	0.36
Disc area	8.53	0.79	<0.001	10.16	1.17	<0.001	8.32	1.00	<0.001	11.93	1.54	<0.001	3.68	0.82	<0.001
Reaction time	−0.02	0.003	<0.001	−0.02	0.004	<0.001	−0.01	0.003	<0.001	−0.02	0.004	<0.001	−0.01	0.003	<0.001
	**(B) Multivariable**														
	**Average RNFL**			**Superior**			**Nasal**			**Inferior**			**Temporal**		
beta	se	p	beta	se	p	beta	se	p	beta	se	p	beta	se	p	
Age	−0.16	0.02	<0.001	−0.27	0.03	<0.001	−0.19	0.02	<0.001	−0.20	0.04	<0.001	—	—	—
Education	—	—	—	—	—	—	—	—	—	—	—	—	—	—	—
SphE	1.18	0.13	<0.001	1.43	0.22	<0.001	1.33	0.16	<0.001	1.84	0.19	<0.001	—	—	—
Disc area	6.03	1.01	<0.001	6.43	1.19	<0.001	5.39	0.98	<0.001	7.70	1.20	<0.001	3.59	0.85	<0.001
Reaction time	−0.01	0.004	0.01	—	—	—	—	—	—	−0.01	0.01	0.01	−0.01	0.003	0.002

Legend: This table shows the results from the univariable (**A**) and multivariable (**B**) analysis. RNFL – retinal nerve fibre layer; IMD – Index of multiple deprivation; SphE – spherical equivalent; IOP – Intraocular pressure; IOPc – IOP corrected for corneal thickness by adding thickness as predictor in the regression; se – standard error, p – p value. Results for the variable with p-value > 0.05 in the stepwise regression are not reported.

**Table 3:** Univariate Heritability estimates of RNFL thickness and cognitive variables.

	average RNFL		Superior quadrant		Nasal quadrant		Inferior quadrant		Temporal quadrant	
	estimate	95% CI	estimate	95% CI	estimate	95% CI	estimate	95% CI	estimate	95% CI
A	0.83	0.66–0.85	0.74	0.58–0.77	0.65	0.60–0.69	0.75	0.72–0.79	0.74	0.63–0.78
C	0.00	0.0–0.0	0.00	0.0–0.0	0.00	0.0–0.0	0.00	0.0–0.0	0.00	0.0–0.0
E	0.17	0.15–0.20	0.26	0.23–0.31	0.35	0.31–0.40	0.25	0.21–0.29	0.26	0.22–0.30
	MMSE score*		Recall*		Reaction time*		Verbal fluency			
	estimate	95% CI	estimate	95% CI	estimate	95% CI	estimate	95% CI		
A	0.32	0.23–0.40	0.19	0.09–0.28	0.44	0.37–0.51	0.48	0.41–0.54		
C	0.00	0.0–0.0	0.00	0.0–0.0	0.00	0.0–0.0	0.00	0.0–0.0		
E	0.68	0.60–0.77	0.81	0.79–0.91	0.56	0.49–0.63	0.52	0.46–0.59		

Legend: This table presents the results from univariate heritability modelling for the different RNFL and cognition parameters. RNFL –retinal nerve fibre layer; A –additive genetic factors; C –common/shared environmental factors; E –unique environmental latent factors; MMSE – mini-mental state examination. ^*^Scores were adjusted for age prior to the heritability analysis.

## References

[b1] Blazer D. G. Cognitive Aging: Progress in Understanding and Opportunities for Action. 17–30 (National Academies Press, 2015). 25879131

[b2] Jones-Odeh E. & Hammond C. J. How strong is the relationship between glaucoma, the retinal nerve fibre layer, and neurodegenerative diseases such as Alzheimer’s disease and multiple sclerosis? Eye 29, 1270–1284 (2015). 26337943 10.1038/eye.2015.158 PMC4815693

[b3] Cvekl A. & Tamm E. R. Anterior eye development and ocular mesenchyme: new insights from mouse models and human diseases. Bioessays 26, 374–386 (2004). 15057935 10.1002/bies.20009 PMC2094210

[b4] London A., Benhar I. & Schwartz M. The retina as a window to the brain—from eye research to CNS disorders. Nat. Rev. Neurol. 9, 44–53 (2013). 23165340 10.1038/nrneurol.2012.227

[b5] Laude A. . Retinal nerve fiber layer thickness and cognitive ability in older people: the Lothian Birth Cohort 1936 study. BMC Ophthalmol. 13, 28 (2013). 23822668 10.1186/1471-2415-13-28 PMC3706226

[b6] van Koolwijk L. M. E. . Association of Cognitive Functioning with Retinal Nerve Fiber Layer Thickness. Investig. Opthalmology Vis. Sci. 50, 4576 (2009). 10.1167/iovs.08-3181 19420335

[b7] Khawaja A. P. . Retinal Nerve Fiber Layer Measures and Cognitive Function in the EPIC-Norfolk Cohort Study. Invest. Ophthalmol. Vis. Sci. 57, 1921–1926 (2016). 27092718 10.1167/iovs.16-19067 PMC4849871

[b8] Hougaard J. L. . Evaluation of heredity as a determinant of retinal nerve fiber layer thickness as measured by optical coherence tomography. Invest. Ophthalmol. Vis. Sci. 44, 3011–3016 (2003). 12824246 10.1167/iovs.02-1090

[b9] van Koolwijk L. M. E. . Genetic Contributions to Glaucoma: Heritability of Intraocular Pressure, Retinal Nerve Fiber Layer Thickness, and Optic Disc Morphology. Investig. Opthalmology Vis. Sci. 48, 3669 (2007). 10.1167/iovs.06-1519 17652737

[b10] Schmoll C. . Reaction time as a measure of enhanced blue-light mediated cognitive function following cataract surgery. Br J Ophthalmol. 95, 1656–9 (2011). 21951571 10.1136/bjophthalmol-2011-300677

[b11] Gao L., Liu Y., Li X., Bai Q. & Liu P. Abnormal retinal nerve fiber layer thickness and macula lutea in patients with mild cognitive impairment and Alzheimer’s disease. Arch. Gerontol. Geriatr. 60, 162–167 (2015). 25459918 10.1016/j.archger.2014.10.011

[b12] Kesler A., Vakhapova V., Korczyn A. D., Naftaliev E. & Neudorfer M. Retinal thickness in patients with mild cognitive impairment and Alzheimer’s disease. Clin. Neurol. Neurosurg. 113, 523–526 (2011). 21454010 10.1016/j.clineuro.2011.02.014

[b13] Paquet C. . Abnormal retinal thickness in patients with mild cognitive impairment and Alzheimer’s disease. Neurosci. Lett. 420, 97–99 (2007). 17543991 10.1016/j.neulet.2007.02.090

[b14] Iseri P. K., Altinaş O., Tokay T. & Yüksel N. Relationship between Cognitive Impairment and Retinal Morphological and Visual Functional Abnormalities in Alzheimer Disease. J. Neuroophthalmol. 26, 18–24 (2006). 16518161 10.1097/01.wno.0000204645.56873.26

[b15] Zhao L., Wang Y., Chen C. X., Xu L. & Jonas J. B. Retinal nerve fibre layer thickness measured by Spectralis spectral-domain optical coherence tomography: The Beijing Eye Study. Acta Ophthalmol. (Copenh.) 92, e35–41 (2014). 10.1111/aos.12240 23981513

[b16] Mok K. H., Lee V. W.-H. & So K. F. Retinal nerve fiber layer measurement of the Hong Kong chinese population by optical coherence tomography. J. Glaucoma 11, 481–483 (2002). 12483090 10.1097/00061198-200212000-00004

[b17] Liu D. . Thinner changes of the retinal nerve fiber layer in patients with mild cognitive impairment and Alzheimer’s disease. BMC Neurol. 15, 14 (2015). 25886372 10.1186/s12883-015-0268-6 PMC4342899

[b18] Shi Z. . Greater attenuation of retinal nerve fiber layer thickness in Alzheimer’s disease patients. J. Alzheimers Dis. 40, 277–283 (2014). 24413621 10.3233/JAD-131898

[b19] Parisi V. . Morphological and functional retinal impairment in Alzheimer’s disease patients. Clin. Neurophysiol. 112, 1860–1867 (2001). 11595144 10.1016/s1388-2457(01)00620-4

[b20] Tatham A. J. . Glaucomatous retinal nerve fiber layer thickness loss is associated with slower reaction times under a divided attention task. Am. J. Ophthalmol. 158, 1008–1017 (2014). 25068641 10.1016/j.ajo.2014.07.028 PMC4515218

[b21] Shen Y. . Retinal nerve fiber layer thickness is associated with episodic memory deficit in mild cognitive impairment patients. Curr Alzheimer Res. 11, 259–266 (2014). 24484274 10.2174/1567205011666140131114418

[b22] Shi Z. . The Utilization of Retinal Nerve Fiber Layer Thickness to Predict Cognitive Deterioration. J Alzheimers Dis JAD. 49, 399–405 (2015). 26484909 10.3233/JAD-150438

[b23] Toth C. A. . A comparison of retinal morphology viewed by optical coherence tomography and by light microscopy. Arch. Ophthalmol. Chic. Ill 1960 115, 1425–1428 (1997). 10.1001/archopht.1997.01100160595012 9366674

[b24] Hinton D. R., Sadun A. A., Blanks J. C. & Miller C. A. Optic-nerve degeneration in Alzheimer’s disease. N. Engl. J. Med. 315, 485–487 (1986). 3736630 10.1056/NEJM198608213150804

[b25] Quigley H. A., Sanchez R. M., Dunkelberger G. R., L’Hernault N. L. & Baginski T. A. Chronic glaucoma selectively damages large optic nerve fibers. Invest. Ophthalmol. Vis. Sci. 28, 913–920 (1987). 3583630

[b26] Singer J. J., MacGregor A. J., Cherkas L. F. & Spector T. D. Genetic influences on cognitive function using the Cambridge Neuropsychological Test Automated Battery. Intelligence 34, 421–428 (2006).

[b27] Guggenheim J. A. . Coordinated genetic scaling of the human eye: shared determination of axial eye length and corneal curvature. Invest. Ophthalmol. Vis. Sci. 54, 1715–1721 (2013). 23385790 10.1167/iovs.12-10560 PMC3626516

[b28] Verhoeven V. J. M. . Genome-wide meta-analyses of multiancestry cohorts identify multiple new susceptibility loci for refractive error and myopia. Nat. Genet. 45, 314–318 (2013). 23396134 10.1038/ng.2554 PMC3740568

[b29] Wood A. R. . Defining the role of common variation in the genomic and biological architecture of adult human height. Nat. Genet. 46, 1173–1186 (2014). 25282103 10.1038/ng.3097 PMC4250049

[b30] Oktem E. O. . The relationship between the degree of cognitive impairment and retinal nerve fiber layer thickness. Neurol. Sci. 36, 1141–1146 (2015). 25575807 10.1007/s10072-014-2055-3

[b31] Bendschneider D. . Retinal Nerve Fiber Layer Thickness in Normals Measured by Spectral Domain OCT. J. Glaucoma 19, 475–482 (2010). 20051888 10.1097/IJG.0b013e3181c4b0c7

[b32] Moayyeri A., Hammond C. J., Valdes A. M. & Spector T. D. Cohort profile: TwinsUK and healthy ageing twin study. Int. J. Epidemiol. dyr207 (2012). 10.1093/ije/dyr207 PMC3600616 22253318

[b33] Folstein M. F., Folstein S. E. & McHugh P. R. ‘Mini-mental state’: a practical method for grading the cognitive state of patients for the clinician. J. Psychiatr. Res. 12, 189–198 (1975). 1202204 10.1016/0022-3956(75)90026-6

[b34] Cummings J. L. Mini-Mental State Examination: norms, normals, and numbers. Jama 269, 2420–2421 (1993). 8479071

[b35] Carcaillon L., Amieva H., Auriacombe S., Helmer C. & Dartigues J.-F. A subtest of the MMSE as a valid test of episodic memory? Comparison with the Free and Cued Reminding Test. Dement. Geriatr. Cogn. Disord. 27, 429–438 (2009). 19401632 10.1159/000214632

[b36] Lezak M. D. Neuropsychological Assessment. (Oxford University Press, 2004).

[b37] Newcombe F. Missile wounds of the brain: A study of psychological deficits. (1969).

[b38] Coslett H. B., Bowers D., Verfaellie M. & Heilman K. M. Frontal verbal amnesia. Phonological amnesia. Arch. Neurol. 48, 949–955 (1991). 1953420 10.1001/archneur.1991.00530210075027

[b39] Deary I. J., Liewald D. & Nissan J. A free, easy-to-use, computer-based simple and four-choice reaction time programme: the Deary-Liewald reaction time task. Behav. Res. Methods 43, 258–268 (2011). 21287123 10.3758/s13428-010-0024-1

